# Insights into lignocellulose degradation: comparative genomics of anaerobic and cellulolytic *Ruminiclostridium*-type species

**DOI:** 10.3389/fmicb.2023.1288286

**Published:** 2023-11-23

**Authors:** Mengcheng You, Qiuyun Zhao, Yuansheng Liu, Wenhao Zhang, Zhewei Shen, Zhenxing Ren, Chenggang Xu

**Affiliations:** ^1^Key Laboratory of Chemical Biology and Molecular Engineering of Ministry of Education, Institute of Biotechnology, Shanxi University, Taiyuan, Shanxi Province, China; ^2^College of Animal Science and Technology & College of Veterinary Medicine, Zhejiang Agriculture and Forestry University, Hangzhou, Zhejiang Province, China; ^3^Institute of Applied Chemistry, Shanxi University, Taiyuan, Shanxi Province, China

**Keywords:** *Ruminiclostridium papyrosolvens*, comparative genomics, pan-genome, CAZymes, cellulosome

## Abstract

Mesophilic, anaerobic, and cellulolytic *Ruminiclostridium*-type bacterial species can secrete an extracellular, multi-enzyme machinery cellulosome, which efficiently degrades cellulose. In this study, we first reported the complete genome of *Ruminiclostridium papyrosolvens* DSM2782, a single circular 5,027,861-bp chromosome with 37.1% G + C content, and compared it with other *Ruminiclostridium*-type species. Pan-genome analysis showed that *Ruminiclostridium*-type species share a large number of core genes to conserve basic functions, although they have a high level of intraspecific genetic diversity. Especially, KEGG mapping revealed that *Ruminiclostridium*-type species mainly use ABC transporters regulated by two-component systems (TCSs) to absorb extracellular sugars but not phosphotransferase systems (PTSs) that are employed by solventogenic clostridia, such as *Clostridium acetobutylicum*. Furthermore, we performed comparative analyses of the species-specific repertoire of CAZymes for each of the *Ruminiclostridium*-type species. The high similarity of their cohesins suggests a common ancestor and potential cross-species recognition. Additionally, both differences between the C-terminal cohesins and other cohesins of scaffoldins and between the dockerins linking with cellulases and other catalytic domains indicate a preference for the location of cellulosomal catalytic subunits at scaffoldins. The information gained in this study may be utilized directly or developed further by genetic engineering and optimizing enzyme systems or cell factories for enhanced biotechnological biomass deconstruction and biofuel production.

## Introduction

Lignocellulose is observed plentifully in nature and is obtained globally, considering it a fascinating source of feedstock for bio-based energy and chemicals. In nature, the direct hydrolysis of lignocellulose is carried out exclusively by microorganisms. Cellulolytic clostridia, such as *Clostridium* (*Hungateiclostridium*) *thermocellum* (Mazzoli and Olson, 2020), *Clostridium* (*Thermoclostridium*) *stercorarium* (Poehlein et al., 2013), *Clostridium* (*Ruminiclostridium*) *cellulolyticum* (Desvaux, 2005), and *Clostridium cellulovorans* (Tamaru et al., 2010, 2011), which are ubiquitous in cellulosic anaerobic environments, represent a major paradigm for efficient biological degradation of cellulosic biomass (Demain et al., 2005; Ransom-Jones et al., 2012). Many of these anaerobes digest cellulose via a cell surface-attached extracellular enzymatic complex called the cellulosome, where primarily catalytic components (including glycoside hydrolases, carbohydrate esterases, and polysaccharide lyases) are integrated onto a non-catalytic macromolecular scaffoldin subunit (Bayer et al., 2004, 2008). The scaffoldins bear modules called cohesin that interact with their modular counterparts, called dockerins, usually conjugated to enzymatic subunits or other scaffoldins. In addition, the scaffoldin may contain a carbohydrate-binding module (CBM) that guides the complex and its intricate set of component enzymes to the surface of the cellulosic substrate (Guillen et al., 2010; Hyeon et al., 2013).

To distinguish them from non-cellulolytic clostridia, these cellulolytic clostridia from Ludwig et al.’s (2019) clostridial cluster III (Garrity et al., 2010; Galperin et al., (2012) were first placed in the new genus “*Ruminiclostridium*” proposed by Yutin et al. (2012), Garrity et al. (2010), Galperin et al. (2012). Whereas the members of clostridial cluster III were further distributed into four new sublineages based on the phylogenetic analysis of 16S rRNA gene sequences made by Zhang X. et al. (2018), including three new genera, *Thermoclostridium*, *Hungateiclostridium*, and *Ruminiclostridium*. Members of *Ruminiclostridium* were mesophilic, spore-forming, cellulosome-producing, and cellulose-degrading bacteria, including *R. cellobioparum*, *R. cellulolyticum*, *R. hungatei*, *R. josui*, *R. papyrosolvens*, *R. termitidis,* and *R. sufflavum*. Their genome size ranged from 4.1 to 6.4 Mb, and the G + C content varied from 36 to 42 mol%. It is noteworthy that they harbor the *cip-cel* gene cluster, which encodes major cellulosomal components that are essential for cellulose degradation. Up to now, 10 whole-genome sequences of *Ruminiclostridium-*type species have been published in NCBI, which allowed us to perform a detailed analysis of the architecture, putative regulation, and evolution of the cellulolytic machinery of mesophilic cellulosome-producing clostridia.

In this study, we first sequenced and completed the genome of *R. papyrosolvens* DSM2782 to understand the mechanism of lignocellulose degradation in mesophilic and cellulolytic clostridia. We further compared it with other nine *Ruminiclostridium*-type species and *C. cellulovorans* 743B, which is involved in mesophilic cellulosome-producing species. Their evolutionary information, genomic diversity, cellulose degradation profiles, and cellulosomal structures were explored using comparative genome analysis. The results of comparative genomics among multiple *Ruminiclostridium*-type species offer new insights into genome evolution involving lignocellulose degradation.

## Materials and methods

### Public genomic resources

The genome and protein sequences of *Clostridium acetobutylicum* ATCC824, *Clostridium cellulovorans* 743B, *Ruminiclostridium* sp. BNL1100, *Ruminiclostridium cellobioparum* DSM1351, *Ruminiclostridium termitidis* CT1112, *Ruminiclostridium cellulolyticum* H10, *Ruminiclostridium herbifermentans* MA18, *Ruminiclostridium hungatei* DSM14427, *Ruminiclostridium josui* JCM17888, *Ruminiclostridium papyrosolvens* C7, *Ruminiclostridium sufflavum* DSM19573, and *Ruminiclostridium papyrosolvens* DSM2782 strains (Supplementary Table S1) were downloaded from the NCBI database^1^ for comparative analysis.

### Culture conditions and DNA extraction of *Ruminiclostridium papyrosolvens* DSM2782

*Ruminiclostridium papyrosolvens* DSM2782 was cultured anaerobically at 35°C in 250-ml flasks with a 100-ml working volume of GS-2 liquid medium (Johnson et al., 1981; Cui et al., 2012) (K_2_HPO_4_ 2.9 g/L, KH_2_PO_4_ 1.5 g/L, urea 2.1 g/L, resazurin 1.0 mg/L, yeast extract 6.0 g/L, cysteine–HCl 0.5 g/L, MOPS 10.0 g/L, and trisodium citrate 3.0 g/L, pH 7.4) supplemented with 3.0 g/L of cellobiose (Yuanye Biotechnology, Shanghai, China). The medium for cultivation was depleted of oxygen in an anaerobic chamber (COY, United States) using resazurin (0.0005% g/L) as the indicator, and then sterilized at 121°C for 20 min. The genomic DNA of *R. papyrosolvens* DSM2782 was extracted using (Illumina, United States) and Template Prep Kit 1.0 (PacBio, United States). The quantity and purity of extracted DNA were determined using a Hou et al. (2021) (Thermo Scientific, United States). The integrity of genomic DNA was further checked by agarose gel electrophoresis to evaluate its quality. DNA was stored at −20°C until use.

### Genome assembly and annotation in *Ruminiclostridium papyrosovlens* DSM2782

The raw sequence data generated from Illumina and PacBio sequencing was utilized for bioinformatics investigation; the whole-genome sequence was assembled using both Illumina and PacBio quality reads. For quality trimming, a value data statistic was used, from which the low-value information could be eliminated to form clean reads (Supplementary Table S2). The reads were then assembled into contigs by the Unicycler (Wick et al., 2017). The final step was completed and finished manually, generating a whole genome with seamless chromosomes. After genome annotation and genes prediction by the NCBI Prokaryotic Genome Annotation Pipeline (PGAP) server (Tatusova et al., 2016), further bioinformatics analysis was performed.

### Comparative genomics

The complete genomes of *R. papyrosolvens* DSM2782 were compared with those of other *Ruminiclostridium* species, *C. acetobutylicum* ATCC824, and *C. cellulovorans* 743B using the BLAST Ring Image Generator (BRIG) (Alikhan et al., 2011) to determine the overall sequence similarity between the strains. Each circular genomic map was drawn using the genome of one reference strain based on a local BLAST+ with standard parameters (50% lower and 70% upper cutoff for identity and an E-value of 1e^−5^). The ring color gradients correspond to varying degrees of the identity of BLAST matches (Altschul et al., 1990). Circular genomic maps also include information on GC skew and GC content, and their evolutionary relationship was inferred by FastTree (Price et al., 2009) using the representative genomes in the Genome Taxonomy Database (GTDB) (Parks et al., 2022) as references. The phylogenetic tree was visualized using the Interactive Tree of Life (iTOL) (Letunic and Bork, 2021).

### Pan-genome and functional analysis

All the protein sequences were calculated usingOrthoFinder2 software with the DIAMOND method to identify homologous groups of protein families in the pan-genome (Emms and Kelly, 2019; Buchfink et al., 2021). The core genome families represented the genes or proteins shared by all 10 *Ruminiclostridium* species. The necessary genome families comprised the genes or proteins shared by at least two strains but not by all 10 *Ruminiclostridium* species. The remaining genes or proteins occurring only in one *Ruminiclostridium* were clustered into unique genome families. To gain more information on the functional characteristics of *Ruminiclostridium-*type species, all the homologous protein sequences were annotated to COG^2^ by using BLASTp (Altschul et al., 1997); a search was performed against the COG database with an E-value cutoff of 1 × 10^−5^. The top of the annotation results was selected as the best annotation for homologous families, and then it was assigned to functional categories.

### Bioinformatics identification of transporter pathways in *Clostridia*

Identification of genes encoding ABC transporter systems and PTSs in 12 *Clostridia* species was performed using BlastKOALA^3^ (Kanehisa et al., 2016b). Additionally, genes encoding TCSs were searched using HMMER 3.0 with TCS characteristic domains from the Pfam database^4^ as references. The sequences were manually sorted to remove the redundancy, and the remaining proteins were considered as identified TCS proteins.

### Annotation of genes involving degradation of lignocellulose

Genes encoding carbohydrate-active enzymes (CAZymes) in the genomes of *Ruminiclostridium* species were predicted based on dbCAN2 (Zhang H. et al., 2018) and classified into families of glycoside hydrolases (GH), carbohydrate esterases (CE), polysaccharide lyases (PL), carbohydrate-binding modules (CBMs), auxiliary activities (AAs), and S-layer homology (SLH) by running a hmmscan of HMMER with an E-value cutoff of 1 × 10^−15^. The Easyfig software (Sullivan et al., 2011) was applied to reveal the homology of the cellulosome gene cluster relationships. Furthermore, cohesin and dockerin modules were predicted using Pfam-supported families as queries. Phylogenetic trees of cohesin and dockerin modules were built with MEGAX software (Kumar et al., 2018).

## Results

### Genome assembly of *Ruminiclostridium Papyrosolvens* DSM2782

In this study, the genome of *R. papyrosolvens* DSM2782 was sequenced and completed by next-generation sequencing (NGS) and third-generation PacBio single-molecule sequencing technology, consisting of a single circular 5,027,861 bp chromosomewith a G + C content of 37.1%. In total, 4,274 coding DNA sequences (CDSs) were predicted, along with 24 rRNA and 62 tRNA genes (GenBank Accession Number CP119677.1). However, 40 SNPs and 200 InDels were identified by comparison with the previous version of the draft genome (GenBank Accession Number ACXX00000000.2) (Hemme et al., 2010). The majority (35) of SNPs were observed in the coding regions, of which 13 variants are nonsense mutations. Almost half of the InDels (95) occurred in polynucleotide regions, which corrected 27 pseudogenes predicted in the previous version (Supplementary Table S3). Thus, the resequencing results revealed that the genome of *R. papyrosolvens* DSM2782 in our laboratory has mutated compared with the original strain.

**Figure 1:**
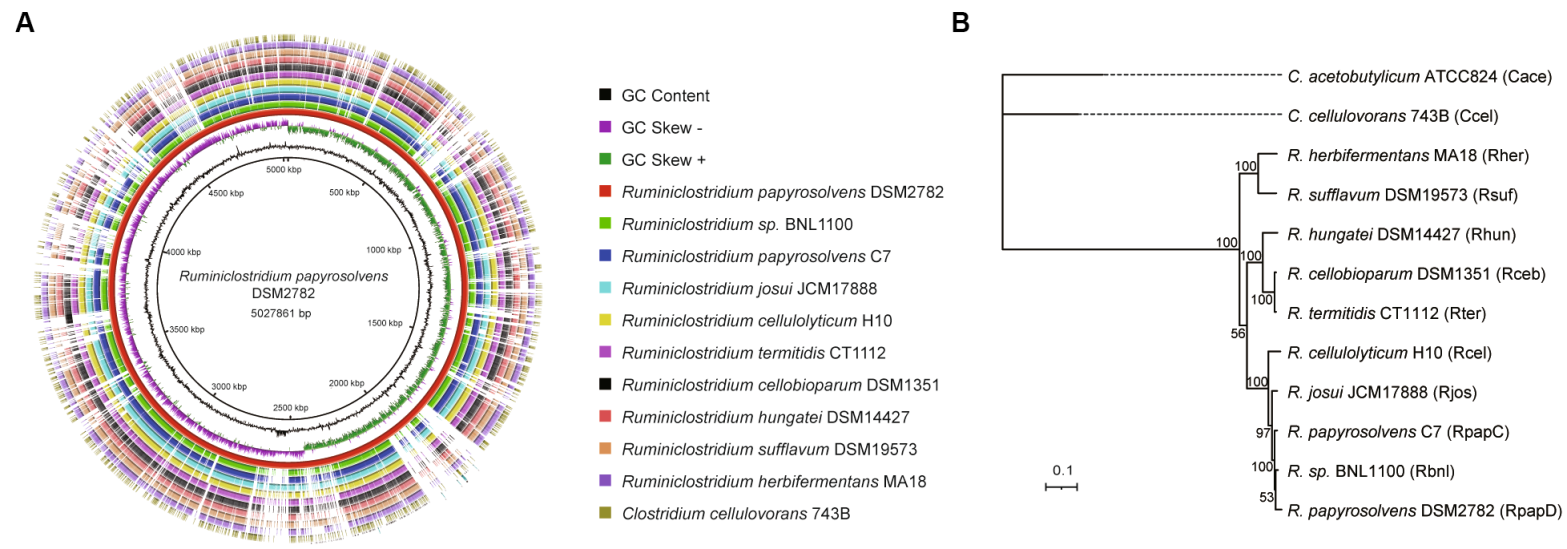
Whole-genome comparisons of *Ruminiclostridium*-type species. **(A)** Whole-genome comparisons, from outer to inner ring: *C. cellulovorans* 743B, *R. herbifermentans* MA18, *R. sufflavum* DSM19573, *R. hungatei* DSM14427, *R. cellobioparum* DSM1351, *R. termitidis* CT1112, *R. cellulolyticum* H10, *R. josui* JCM17888, *R. papyrosolvens* C7, *R.* sp. BNL1100; reference genome: *R. papyrosolvens* DSM2782. The color intensity in each ring represents the BLAST match identity. **(B)** Phylogenetic tree of mesophilic cellulolytic clostridia using the representative genomes in the Genome Taxonomy Database (GTDB) as references. Bootstrap values on nodes are indicated by >50. Bar 0.1 represents the nucleotide substitutions per position.

Furthermore, the complete genome of *R. papyrosolvens* DSM2782 was compared with that of other *Ruminiclostridium***-**type species (*R. papyrosolvens* C7, *R.* sp. BNL1100, *R. josui* JCM17888, *R. cellulolyticum* H10, *R. termitidis* CT1112, *R. cellobioparum* DSM1351, *R. hungatei* DSM14427, *R. sufflavum* DSM19573, and *R. herbifermentans* MA18) and *C. cellulovorans* 743B using BLAST Ring Image Generator (BRIG) (Figure 1A). It indicated that most regions in the test strains show an identity higher than 70% of the alignment reference genome of *R. papyrosolvens* DSM2782. There is a position with higher GC content in the region ranging from 2,500 to 2,600 kbp, which harbors several genes encoding SDR family NAD(P)-dependent oxidoreductases. Meanwhile, several gaps highlighting the missing regions are visible at positions of 700–800, 1800–1900, and 2000–2,100 kbp, where genes encoding the ABC transporter system and different types of domain-containing proteins are enriched (Figure 1A). Finally, based on the BRIG analysis, the result represents a snapshot of the genetic diversity and close relatedness of the bacteria which belong to the Clostridia group.

Moreover, a phylogenetic analysis of *Ruminiclostridium***-**type species was performed by GTDB-Tk (Chaumeil et al., 2022) based on whole-genome sequences compared with *C. cellulovorans* 743B and *C. acetobutylicum* ATCC824, which are mesophilic cellulosome-producing clostridia but not belong to *Ruminiclostridium*-type species (Figure 1B). It showed that *Ruminiclostridium* species are phylogenetically distant from the *Clostridium* species of *C. cellulovorans* 743B and *C. acetobutylicum* ATCC824 and can be regarded as three clades supported by high bootstrap BP values. It was uncovered that *R. papyrosolvens* DSM2782 is closely related to *Ruminiclostridium* species BNL1100, *R. papyrosolvens* C7, *R. josui* JCM17888, and *R. cellulolyticum* H10 and belonged to the same clade, which had the farthest relationship with clostridia species.

### Pan-genome of *Ruminiclostridium*-type species

**Figure 2:**
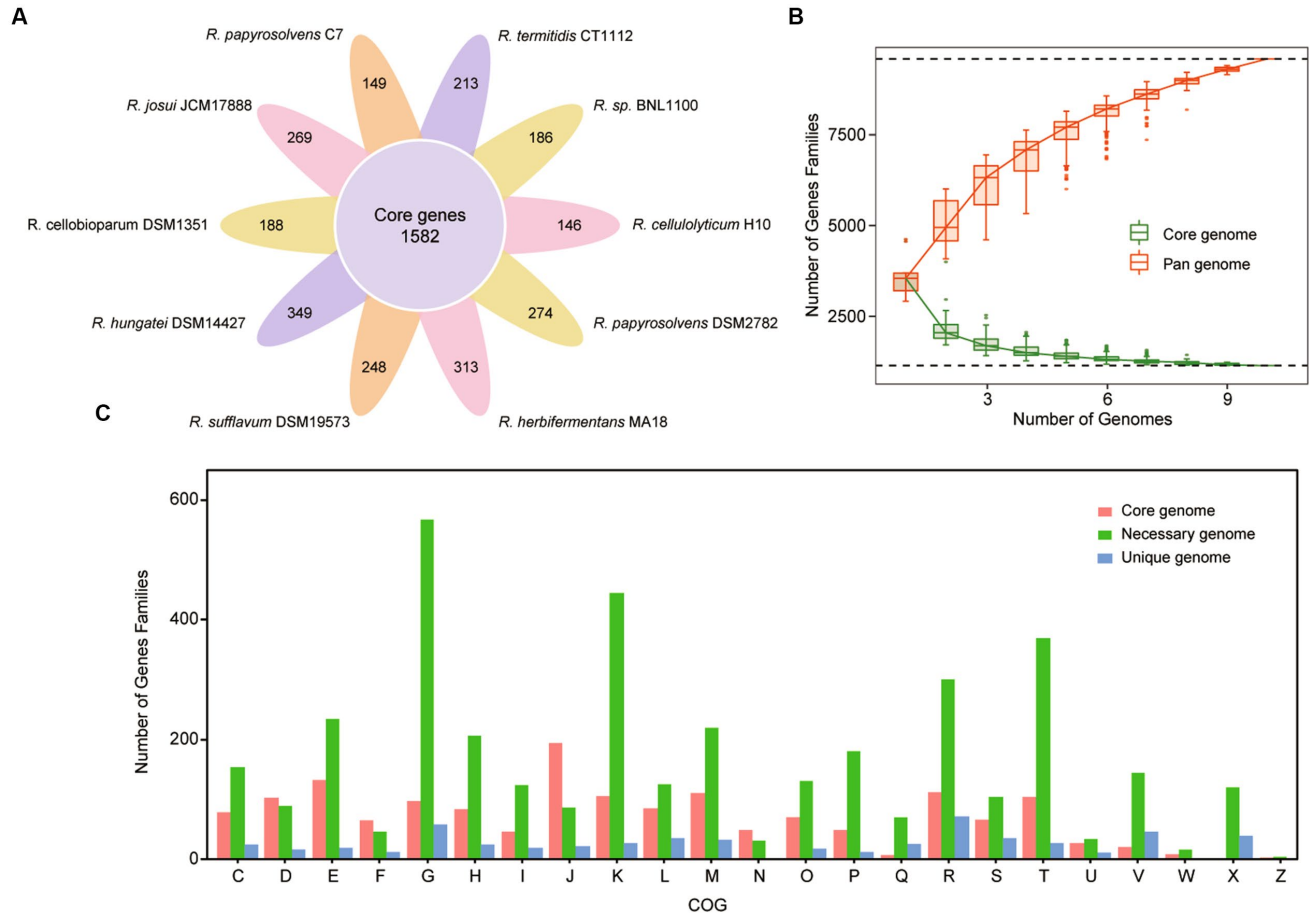
Pan-genome structure and gene functional distribution of *Ruminiclostridium*-type species. **(A)** Core and unique gene families in each *Ruminiclostridium* species. The number of core genomes shared by all species is in the center (1,582). **(B)** The size of the pan-genome (red) and core genome (green) shared by different *Ruminiclostridium* species. **(C)** Distribution of the Clusters of Orthologous Groups (COG) categories in *Ruminiclostridium* core, necessary, and unique genes (C, energy production and conversion; D, Cell cycle control, cell division, chromosome partitioning; E, amino acid transport and metabolism; F, nucleotide transport and metabolism; G, carbohydrate transport and metabolism; H, coenzyme transport and metabolism; I, lipid transport and metabolism; J, translation, ribosomal structure and biogenesis; K, transcription; L, replication, recombination, and repair; M, cell wall/membrane/envelope biogenesis; N, cell motility; O, posttranslational modification, protein turnover, chaperones; P, inorganic ion transport and metabolism; Q, secondary metabolites biosynthesis, transport, and catabolism; R, general function prediction only; S, function unknown; T, signal transduction mechanisms; U, intracellular trafficking, secretion, and vesicular transport; V, defense mechanisms; W, extracellular structures; X, mobilome: prophages, transposons; Z, cytoskeleton).

To capture the entire genomic diversity of these mesophilic and cellulolytic *Ruminiclostridium* species, we performed a pan-genome analysis (Tettelin et al., 2008; Lee et al., 2021). It showed that the total genomes of 10 *Ruminiclostridium*-type species included 41,055 proteins with 8,414 orthologous gene families, of which 1,582 orthologous gene families were shared by all 10 analyzed genomes regarded as the core genome (Figure 2A). The necessary genome sharing by at least two species and the unique genome found in only one strain included 4,497 and 2,335 gene families, respectively. Among these mesophilic and cellulolytic *Ruminiclostridium*-type species, *R. hungatei* DSM14427 possessed more unique genes, with 349 genes (Figure 2A). The variable genes account for approximately 81.20% of the whole pan-genome, signifying the high level of genetic diversity in the members of mesophilic and cellulolytic *Ruminiclostridium-*type species.

Furthermore, the curves of the core genome and pan-genome size of these genomes with the increase in the number of genomes showed that the pan-genome size increased almost exponentially with the number of genomes, while the core genome size was being narrowed (Figure 2B). When the number of added genomes reached 10, the size of the pan-genome still increased. According to Figure 2B, the red line represented pan-genome, it’s increased. The measured size of the pan-genome was well fitted with a power law function (y = Ax^b^, where A is 3648.8 and b is 0.3785), suggesting that the pan-genome might still be influenced by the inclusion of new genome sequences. The complete pan-genome of the *Ruminiclostridium* genus is likely to be substantially larger than that estimated by these 10 genomes. Thus, although the mesophilic and cellulolytic *Ruminiclostridium* species have a high level of intraspecific genetic diversity, their core genes have a stronger tendency to conserve basic functions.

Moreover, to clarify the functional characteristics of the *Ruminiclostridium* species genome, an analysis of the clusters of orthologous groups (COGs) was performed (Galperin et al., 2015). The functional categories of the genes were assigned to core, necessary, and unique classes, and the results showed that the gene families in the *Ruminiclostridium* core genome were enriched for genes involved in “translation, ribosomal structure, and biogenesis” (Figure 2C). The overall proportion of genes involved in “translation, ribosomal structure, and biogenesis” in the core genome was 11.9% (194/1,620), whereas that in the necessary and unique genomes was 2.28% (87/3,807) and 3.75% (22/586), respectively. Therefore, the COG analysis results highlighted that more core genes perform fundamental housekeeping functions than necessary and unique genes.

### Transmembrane transport systems

**Figure 3:**
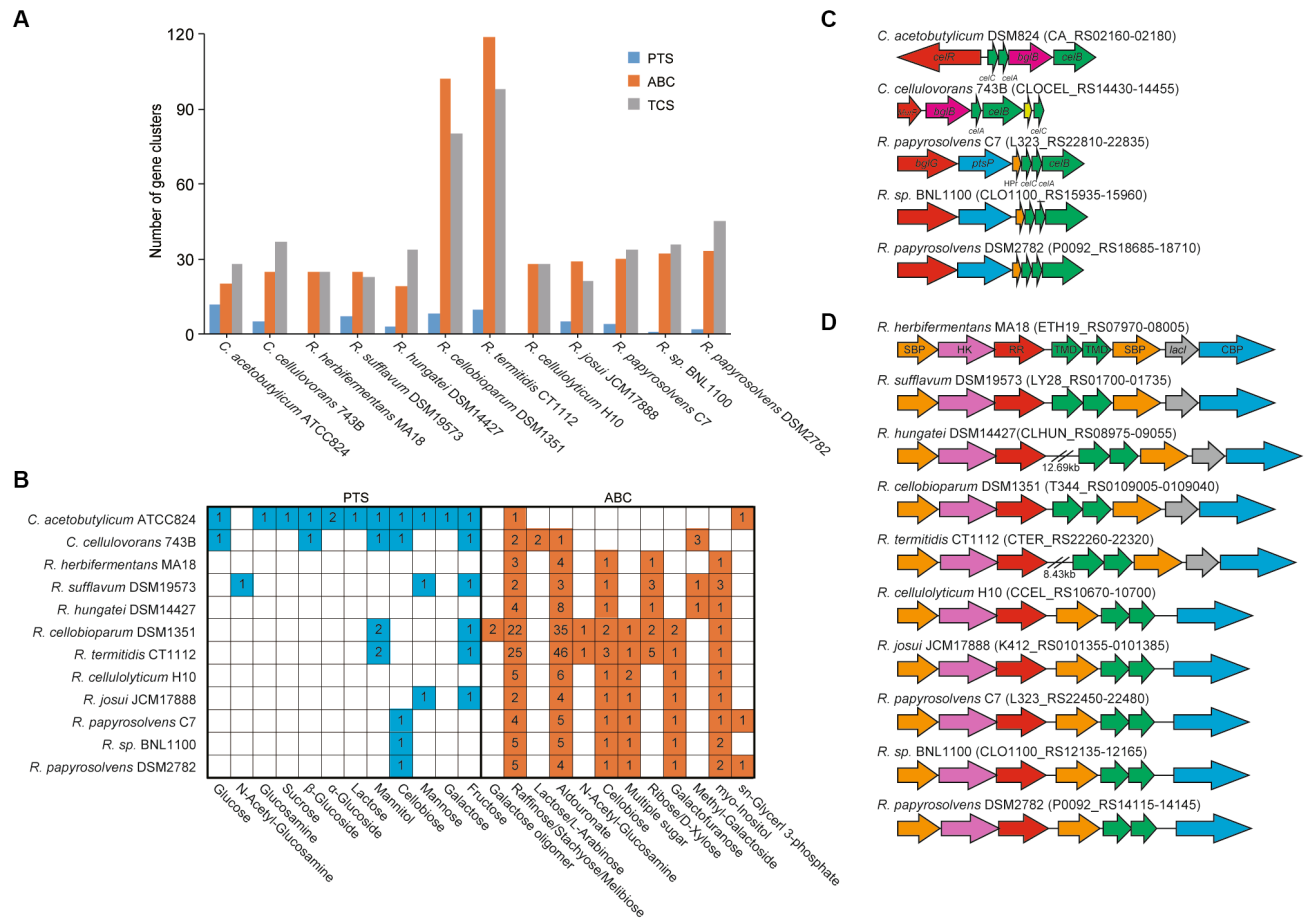
ABC transport systems and PTSs in *Ruminiclostridium*-type species compared with *C. acetobutylicum* and *C. cellulovorans*. **(A)** Comparsion of the number of gene clusters encoding ABC transporters, PTSs, and TCSs. **(B)** The number of ABC transporters and PTSs involved in the transportation of various sugars. **(C)** The gene clusters encoding PTSs and **(D**) ABC transport systems for cellobiose transportation. Soulte-binding protein (SBP), histidine kinase (HK), response regulator (RR), transmembrane domain (TMD), and cellobiose phosphorylase (CBP).

We further predicted and compared the transporters, including phosphoenolpyruvate-dependent phosphotransferase systems (PTSs), ATP-binding cassette (ABC) transporters, and TCSs, among these cellulolytic clostridia (Dassa and Bouige, 2001; Tian et al., 2017; Cheng et al., 2021), which the cellulolytic bacteria usually use two-component systems (TCSs) to sense extracellular sugars and regulate the expression of transporters and CAZymes (Joseph et al., 2002; Xu et al., 2013). It showed that the number of ABC transporters in each genome was similar to that of TCSs but was more than that of PTSs. Meanwhile, the number of ABC transporters and TCSs appeared to be positively related to the size of genomes. For example, the number of ABC transporters and TCSs in *R. cellobioparum* and *R. termitidis* was more than twice as much as that of other clostridia, and their genome size was more than 6 Mb, surpassing that of other clostridia (Figure 3A, Supplementary Table S4).

Furthermore, the transportation of various sugars in *Ruminiclostridium*-type species predicted by the KEGG database (Kanehisa et al., 2016a) was compared with that of *C. acetobutylicum* and *C. cellulovorans*. It was indicated that the number of ABC transporters for sugars in *Ruminiclostridium*-type species was much higher than in PTSs (Voigt et al., 2014), confirming that *Ruminiclostridium* species mainly employ ABC transporters to absorb extracellular sugars (Xu et al., 2013; Fosses et al., 2017). Specifically, *R. cellulolyticum* and *R. herbifermentans* harbor no PTSs for sugars. However, it is the exact opposite of *C. acetobutylicum*, in which there are 12 PTSs and only 2 ABC transporters for sugars. As for *C. cellulovorans*, it has 5 PTSs and 8 ABC transporters for the importation of sugars (Figure 3B, Supplementary Table S5), suggesting that PTSs are as important as ABC transporters for the importation of sugars in *C. cellulovorans*.

Moreover, the function of ABC and PTS transporters encoded in genomes was first annotated based on the KEGG database (map02010 for ABC transporters and map02060 for PTSs). The putative transporters involved in sugars in *Ruminiclostridium*-type species were compared with those of *C. acetobutylicum* and *C. cellulovorans*. *C. acetobutylicum* and *C. cellulovorans* harbor a cellobiose PTS (Figures 3B,C), while all *Ruminiclostridium*-type species harbor an orthologous cellobiose ABC transporter that is regulated by its upstream TCS (Fosses et al., 2017) (Figures 3B,D). However, in addition to the ABC transporter, *R. papyrosolvens* C7 and DSM2782, and *Ruminiclostridium* sp. BNL1100, the farthest relationship between *Ruminiclostridium* and *C. acetobutylicum* also evolves an orthologous cellobiose PTS that is not homologous with that of *C. acetobutylicum* and *C. cellulovorans*, which is potentially regulated by its upstream BglG-type transcriptional regulator (Figures 3B,C) (Tangney and Mitchell, 2007).

### CAZyme annotation and distribution

**Figure 4:**
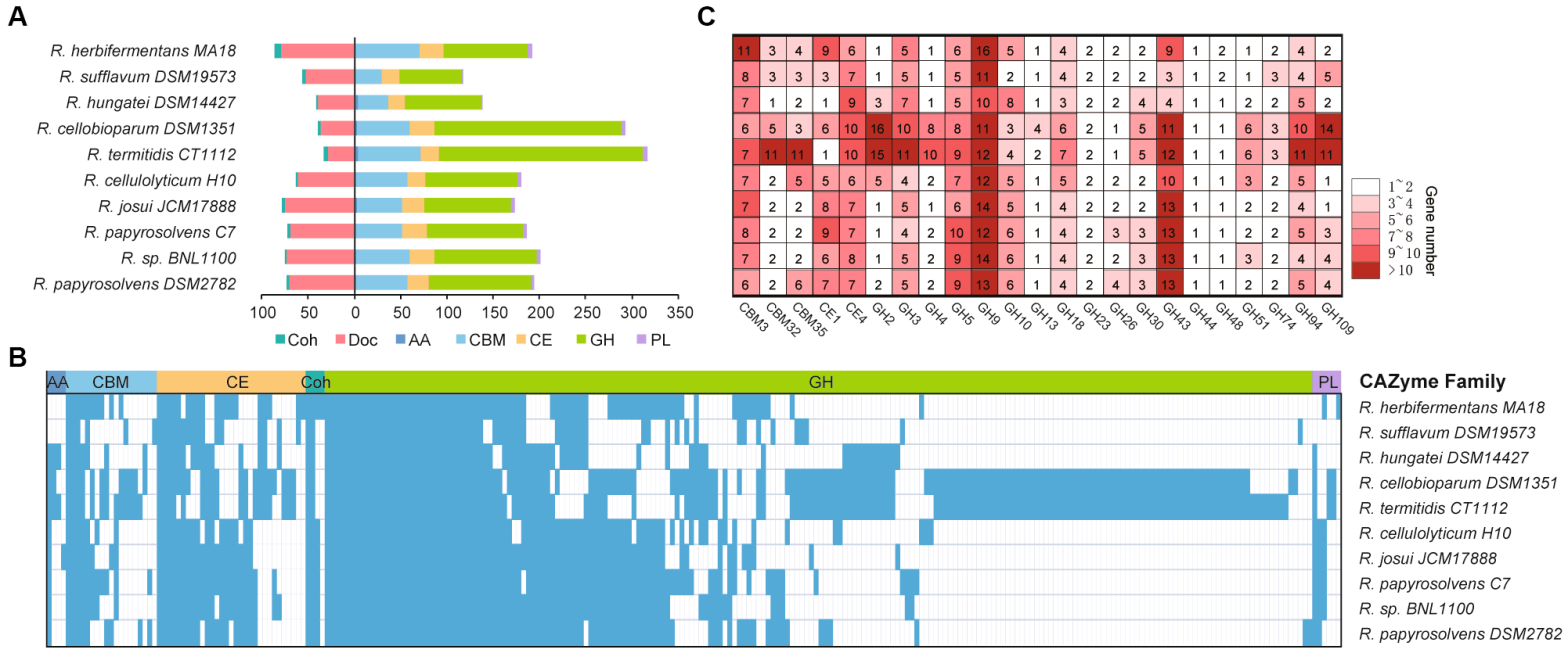
Statistical analysis of CAZymes modules in *Ruminiclostridium*-type species. **(A)** The number of CAZymes is denoted for each genome of a *Ruminiclostridium*-type species. **(B)** Presence (color)/absence (blank) pattern of CAZyme orthologous families in each *Ruminiclostridium*-type species. Glycoside hydrolases (GHs), polysaccharide lyases (PLs), carbohydrate esterases (CEs), carbohydrate-binding modules (CBMs), cohesins (Coh), dokerins (Doc). **(C)** A detailed count of the major CAZyme families.

To understand the complex functions of carbohydrate degradation, carbohydrate-active enzymes (CAZymes) and cellulosomal subunits were predicted and compared among *Ruminiclostridium*-type species. *R. papyrosolvens* DSM2782 harbors 203 putative CAZymes, including one auxiliary activity (AA), 56 carbohydrate-binding modules (CBMs), 23 carbohydrate esterases (CEs), 111 glycoside hydrolases (GHs), and three polysaccharide lyases (PLs). It also has 73 putative cellulosomal subunits which contain cohesin (3) and dockerin domains (70). The number of CAZymes in *R. papyrosolvens* DSM2782 is similar to that of *R. herbifermentans* MA18, *R. cellulolyticum* H10, *R. josui* JCM17888, *R. papyrosolvens* C7, and *Ruminiclostridium* sp. BNL1100, which is higher than *R. sufflavum* DSM19573 and *R. hungatei* DSM14427 but much lower than *R. cellobioparum* DSM1351 and *R. termitidis* CT1112. However, *R. cellobioparum* DSM1351 and *R. termitidis* CT1112 harbor the largest number of CAZymes (298 and 322) but feature the least portfolio of cellulosomal subunits (12 and 11) (Figure 4A, Supplementary Table S6).

Phylogenetic analysis of CAZymes revealed that these 10 *Ruminiclostridium* species included 252 orthologous CAZyme families, with 42 orthologous families shared by all strains. In particular, most of the CAZymes (210) in *R. papyrosolvens* DSM2782, *R. cellulolyticum* H10, *R. josui* JCM17888, *R. papyrosolvens* C7, and *Ruminiclostridium* sp. BNL1100 were orthologous (38.6, 34.3, 35.2, 42.4, and 39.5% of all CAZymes, respectively), while both *R. cellobioparum* DSM1351 and *R. termitidis* CT1112 shared the most orthologous CAZyme families (Figure 4B). This was consistent with the evolutionary relationship of *Ruminiclostridium* species determined by genome alignment (Figure 1B). Furthermore, we counted the number of each CAZyme family to determine the major enzymes for lignocellulose degradation in *Ruminiclostridium*-type species. It was revealed that CAZymes belonging to 3 CBM families, 2 CE families, and 18 GH families are shared in all *Ruminiclostridium*-type species, such as CBM3, GH9, GH43, GH5, GH94 and CE4 families with the largest number (Figure 4C), suggesting that these CAZyme families are critical to cellulose degradation.

### Genes encoding cellulosomal catalytic subunits

**Figure 5:**
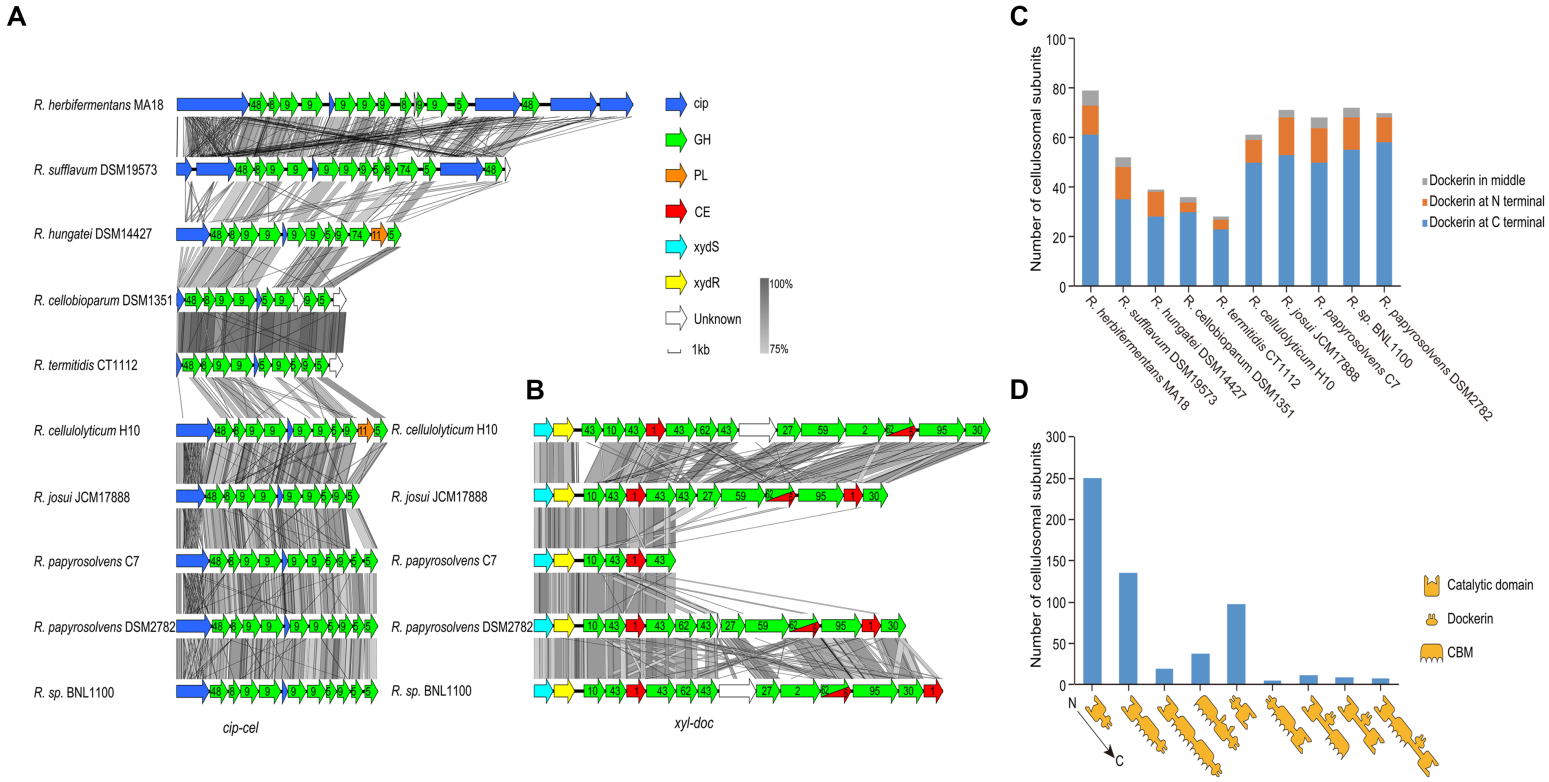
Gene clusters and architectures of catalytic subunits of cellulosomes. **(A,B)** Linear comparisons of the *cip-cel* and *xyl-doc* gene clusters from mesophilic *Ruminiclostridium*-type species. The major scaffoldin gene is represented by *cip*, and CAZymes are denoted by their family numbers. **(C)** Number of cellulosomal subunits with different locations of dockerin domains for each species. **(D)** Statistical analysis of catalytic subunits of cellulosomes with various architectures.

For profiling the cellulosomal system of each genome, we focused on its two gene clusters, *cip-cel* and *xyl-doc* (Maamar et al., 2006; Xu et al., 2013), and the organization of the cellulosomal protein modules. We first performed the homology analysis for the *cip-cel* (Figure 5A) and *xyl-doc* (Figure 5B) gene clusters by using Easyfig. This revealed that both gene clusters have high similarity among various *Ruminiclostridium-*type species. However, the similarities of *cip-cel* gene clusters between *R. herbifermentans* MA18 and *R. sufflavum* DSM19573 and between *R. termitidis* CT1112 and *R. cellobioparum* DSM1351 were higher than those of others, which were consistent with their phylogenetic tree of genomes (Figure 1B).

The *cip-cel* operon exists in all the examined species and is critical for cellulose degradation. The major scaffoldin gene, termed *cip* (Tomazetto et al., 2016), is the first gene, followed by 10 to 16 genes coding for cellulolytic enzymes from the GH 48, GH9, and GH5 families (Morag et al., 1991; Zverlov et al., 2005; Ravachol et al., 2014). In between the genes of the cluster lies a conserved gene, termed *orfX*, which codes for a cohesin-containing protein (Figure 5A) (Doi and Kosugi, 2004). The overall gene organization of the cluster is comparable to that of all species, suggesting that the cellulosomes of the mesophilic *Ruminiclostridium* species originated from a common ancestor. Nevertheless, we still observed two patterns of gene architecture among the different bacteria. *R. herbifermentans* MA18 and *R. sufflavum* DSM19573 harbor more scaffoldin genes in the *cip-cel* cluster than other *Ruminiclostridium*-type species. Both *R. herbifermentans* and *R. sufflavum* have another copy of the scaffoldin and GH48 genes downstream of the general *cip-cel* cluster (Figure 5A).

In addition to the *cip-cel* operon, another gene cluster encoding exclusively secreted dockerin-containing hemicellulases, termed *xyl-doc*, was found in four *Ruminiclostridium* species, which had the farthest relationship with species in clostridia, suggesting that *xyl-doc* evolved later than *cip-cel*. In the upstream of this gene cluster, there are two genes encoding the two-component system (TCS) (Figure 5B).

Furthermore, we analyzed the module organization of the cellulosomal catalytic subunits harboring dockerin domains (576) from all *Ruminiclostridium*-type species. These subunits include three modules: the catalytic domain (CD), CBM, and dockerin (Doc). The majority of dockerins (76.9%) were found at the C-terminal of catalytic subunits, followed by the N-terminal (18.1%) and the middle (5.0%) (Figure 5C). Specifically, the modular structures of catalytic subunits harboring C-terminal dockerins mainly included CD-Doc (250), CD-CBM-Doc (135), CD-CBM-CBM-Doc (19), and CBM-CD-Doc (38), while those of N-terminal dockerins are Doc-CD (98) and Doc-CBM-CD (5). Subunits harboring the middle dockerins contain three modular structures: CD-Doc-CBM (11), CD-Doc-CD (9), and CD-CBM-Doc-CD (8) (Figure 5D). Thus, the modular structures of catalytic subunits mainly have CD-Doc, CD-CBM-Doc, and Doc-CD, which account for 83.9% of the total.

### Phylogenetic relationships between the cohesins and dockerins

**Figure 6:**
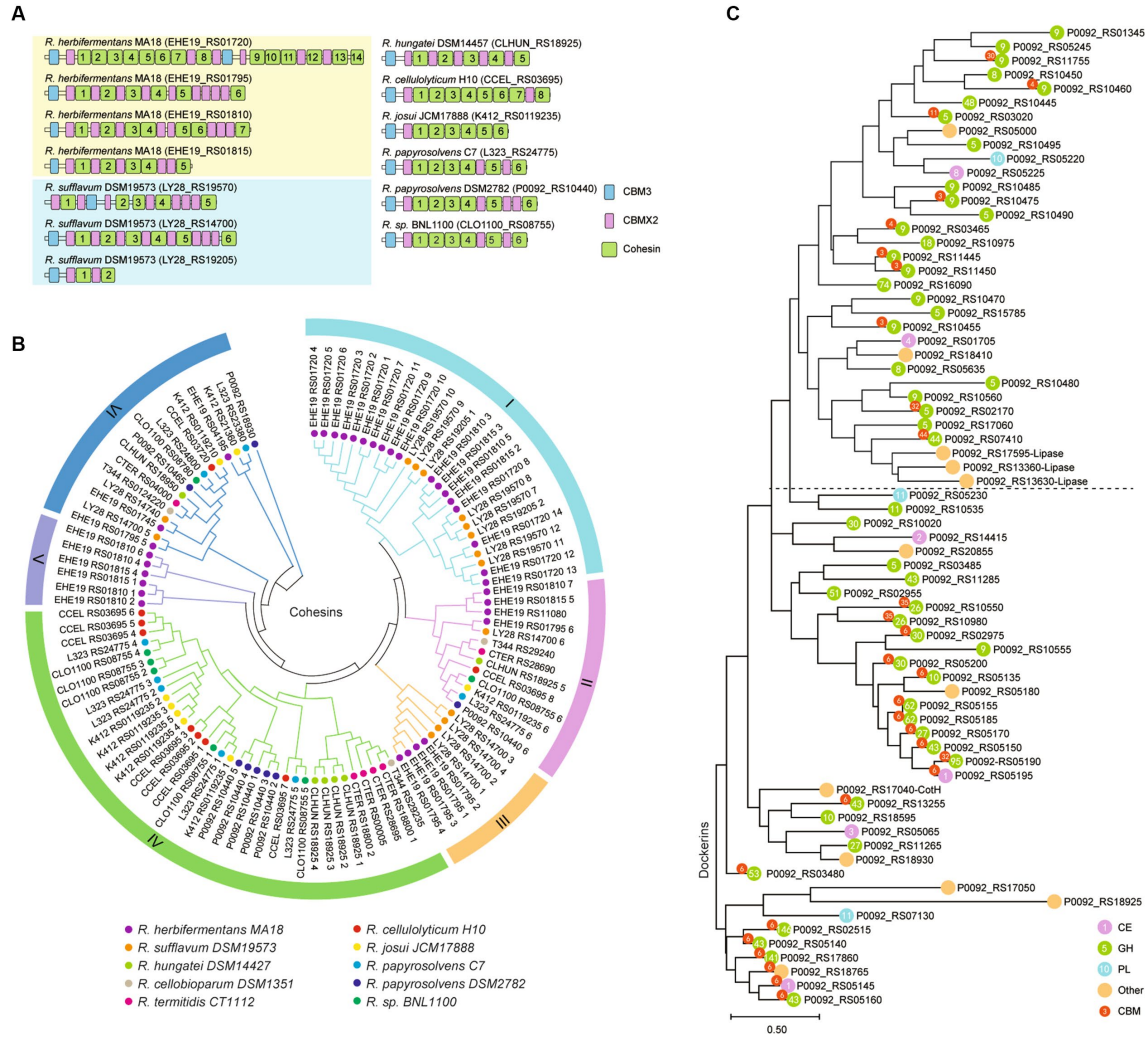
Phylogenetic relationship of dockerin and cohesin modules. **(A)** Modular and domain architectures of the primary scaffoldins of *Ruminiclostridium-*type species. **(B)** Phylogenetic analysis of cohesin domains from 10 *Ruminiclostridium*-type species using MEGAX. Six major branches are labeled with different colors. **(C)** Phylogenetic tree of all dockerins derived from catalytic subunits of the cellulosome in *R. papyrosolvens* DSM2782. The tree was built through 100 bootstraps using a maximum composite likelihood approach based on neighbor-joining algorithms. Bar 0.50 represents the nucleotide substitutions per position.

*Ruminiclostridium*-type species usually harbor a large cellulosomal scaffoldin, but *R. herbifermentans* and *R. sufflavum,* respectively, have four and three scaffoldins. The cellulosomal scaffoldins of *Ruminiclostridium*-type species mainly include cohesin domains interacting with dockerin domains of enzymes, CBM3s binding to cellulose, and CBMX2 binding to cellulose and bacterial cell walls (Poole et al., 1992; Mosbah et al., 2000). The CBM domains are always located at the N-terminal of scaffoldins, except for those encoded by LY28_RS19570. They are followed by cohesin domains ranging in number from two 2 to 14, some of which are separated by CBM X2 (Figure 6A). To determine the difference between cohesins from various *Ruminiclostridium*-type species, the phylogenetic relationship of cohesins was analyzed. A total of 105 cohesins were classified into six groups. It was found that the cohesins between *R. herbifermentans* MA18 and *R. sufflavum* DSM19573 are closely related and are mainly clustered into Groups I, III, and V, while the cohesins from the other eight *Ruminiclostridium*-type species were mainly clustered into Group IV. However, the cohesins located at the C terminal of scaffoldins were separately clustered into Group II (Figure 6B). Thus, it was suggested that cohesins have interspecific similarity and intraspecific dissimilarity among *Ruminiclostridium*-type species, and the intraspecific dissimilarity is related to the location of scaffoldin.

In addition, we used the dockerins of *R. papyrosolvens* DSM2782 as an example to analyze the link between dockerins and the catalytic domains they belong to. Phylogenetic analysis of dockerins of *R. papyrosolvens* DSM2782 showed that the dockerins fused with CBM3 and GH5, GH8, GH9, and GH48 families and others involved in cellulose degradation were clustered at the same clade in the evolutionary tree. The dockerins derived from cellulosomal subunits encoded by the *cip-cel* operon also belong to this clade. However, the other dockerins linked with CBM6 and GH10, GH26, GH30, and GH43 families, including enzymes encoded by the *xyl-doc* gene cluster and others involved in hemicellulose degradation, were mainly grouped into three clades (Figure 6C). It is suggested that the dockerin domain may co-evolve with its linking catalytic domain. Thus, the location of cellulosomal subunits in scaffoldin may not be random, but rather preferential due to the sequence dissimilarity of cohesins and dockerins, as indicated by the scaffoldin locations and the function of the fused catalytic domains.

## Discussion

The ability to grow on and ferment lignocellulose into valuable end products, such as ethanol, makes *Ruminiclostridium*-type species attractive and a potential candidate for biofuel production via consolidated bioprocessing. Understanding the composition and structure of CAZymes and cellulosomes associated with lignocellulose degradation is important for improving our understanding of cellulolytic physiology and identifying engineering targets for improving biomass biofuel production.

### *Ruminiclostridium*-type species are homologous but different

*Ruminiclostridium papyrosolvens* is one of the most highly evolved species among *Ruminiclostridium*-type species (Zou et al., 2018). We have developed a suitable genetic manipulation system in *R. papyrosolvens* (Ren et al., 2019; Wang et al., 2023) and analyzed its secretome in our previous studies, suggesting that it is a potential chassis cell for producing cellulosic biofuels, but further research is limited due to its incomplete genetic information. Thus, the genome of *R. papyrosolvens* DSM2782 was sequenced, completed, and compared with other mesophilic and cellulolytic clostridia in this study. It was revealed that *R. papyrosolvens* DSM2782 is closely related to *Ruminiclostridium* sp. BNL1100, *R. papyrosolvens* C7, *R. josui* JCM17888, and *R. cellulolyticum* H10. They are similar with respect to the composition of CAZymes, the transportation of sugars derived from lignocelluloses, and the gene clusters encoding the cellulosome.

Although *C. cellulovorans* is also an anaerobic, mesophilic, and cellulolytic *Clostridium* harboring the *cip-cel* cluster, it is phylogenetically distant from *Ruminiclostridium*-type species, suggesting that *C. cellulovorans* evolved in a different direction from *Ruminiclostridium*. For example, *C. cellulovorans* employs the PTS for the uptake of cellobiose and cellodextrin that derive from cellulose, while *Ruminiclostridium*-type species mainly transport them through ABC transporters, suggesting that PTSs are as important as ABC transporters for the importation of sugars in *C. cellulovorans*. Thus, *Ruminiclostridium*-type species, *C. acetobutylicum,* and *C. cellulovorans* evolve different strategies to import sugars (Servinsky et al., 2010; Wu et al., 2022). It seems that clostridia evolve more and more ABC transporters to import extracellular sugars as the ability of lignocellulose degradation increases. Additionally, the *cip-cel* cluster in *C. cellulovorans* does not contain a GH8 gene that is conserved in all *Ruminiclostridium*-type species and is shorter than that of *Ruminiclostridium*-type species (Tamaru et al., 2011).

*Ruminiclostridium cellobioparum* and *R. termitidis*, respectively, isolated from the rumen of cattle and the gut of termites (Lal et al., 2013; Mukherjee et al., 2017), have a close phylogenetic relationship. They have the largest genome size (6.13 and 6.42 Mb) among the *Ruminiclostridium* species considered in this study, harboring significantly higher CAZymes than other *Ruminiclostridium* strains. Especially, GH families are spread across more than 50 families in these two strains, including many characteristic/unique GH families, such as GH2, GH3, GH4, and GH51 hemicellulases, GH94 phosphorylases, and GH109 N-acetylhexosaminidase, highlighting the potential ability to produce a wide variety of enzymes needed to breakdown different types of complex biomass components. Considering the open state of the *Ruminiclostridium* pan-genome, the results of the COG enrichment analysis of *Ruminiclostridium* necessary genes and unique genes, especially “carbohydrate transport and metabolism” (G), “transcription” (K), “signal transduction mechanisms” (T), “general function prediction only” (R), and “cell wall/membrane/envelope biogenesis” (M), were consistent with the perspective that larger genomes tend to accumulate functions to enable organisms to achieve a higher degree of ecological diversification. However, *R. cellobioparum* and *R. termitidis* possess the least number of cellulosomal subunits among *Ruminiclostridium* species, which may limit their cellulose degradation capacity, suggesting that there may be abundant non-cellulosic polysaccharide in their living surroundings–animal guts.

Intriguingly, *R. herbifermentans* and *R. sufflavum*, phylogenetically distant from other *Ruminiclostridium*-type species, harbor more scaffoldin genes in the *cip-cel* cluster than other *Ruminiclostridium*-type species. Both *R. herbifermentans* and *R. sufflavum* have another copy of the scaffoldin and GH48 genes downstream of the *cip-cel* cluster. In addition, *R. herbifermentans* still has two scaffoldin genes following the copy of the GH 48 gene, while *R. sufflavum* has another shorter *cip* gene upstream of the *cip-cel* cluster. However, all these encoded scaffoldins in the *cip-cel* clusters are the primary scaffoldins incorporating enzymes, unlike the multiple scaffoldin gene clusters in *H. thermocellum*, which also encode the anchoring scaffoldins that contain type-II cohesins for attachment to the cell surface of the corresponding number of primary scaffoldins (Dassa et al., 2012). Thus, the multiple scaffoldin genes in the *cip-cel* cluster in *R. herbifermentans* and *R. sufflavum* may be the result of a gene duplication event.

### Interaction between cohesins and dockerins is potentially selective

The phylogenetic relationship of cohesins showed interspecific similarity among *Ruminiclostridium*-type species, indicating a general cross-species interaction between their scaffoldins and enzyme subunits in nature, and would imply their conservation in the same ecological niche. However, their putative conserved recognition residues are different from those of the complex cellulosomes from *A. thermocellus*, *B. cellulosolvens*, *R. clariflavum,* and *R. flavefaciens* (Dassa et al., 2017), suggesting that the cohesins of *Ruminiclostridium*-type species cannot exhibit cross-type recognition of the dockerins of complex cellulosomes, but the dockerin of *A. thermocellus* XynB is an exception, which is recognized by the cohesin from *R. cellulolyticum* (Haimovitz et al., 2008). On the other hand, the phylogenetic relationship between cohesins and dockerins showed that the cohesins located at the C-terminal of scaffoldins were significantly different from cohesins at other locations of scaffoldins, and dockerins linking with cellulases were clustered into a distinct clade. This may suggest the location preferences of catalytic subunits in scaffoldins for the best synergistic effect. It has been proven in the minimal cellulosome of *Clostridium saccharoperbutylacetonicum,* in which scaffoldin has only two cohesins (named Coh1 and Coh2). These two cohesins exhibited remarkably different binding patterns. Coh1 presented varied affinities toward the dockerin-containing enzymes, whereas Coh2 was much less selective and exhibited higher affinity toward all dockerins (Levi Hevroni et al., 2020).

Hydrolysis of cellulosic substrates is a major biotechnological challenge. Reconstitution of the biological principle of native cellulosomes may provide a basis for improved cellulolytic activity. In this study, we first sequenced and completed the genome of *R. papyrosolvens* DSM2782, which represented a single circular chromosome (5,027,861 bp, 37.1% G + C content), containing 4,407 coding DNA sequences and 90 RNA-coding genes. Comparative genome analysis showed that *Ruminiclostridium*-type species share a large number of core genes to conserve basic functions, such as the transcription of extracellular sugars and degradation of lignocelluloses, although they have a high level of intraspecific genetic diversity. However, some variations in the number and organization of genes encoding CAZymes and cellulosomes were found. Our analyses, described here, contribute to the understanding of the variety and similarity of genomes in *Ruminiclostridium*-type species involved in lignocellulose degradation. They should help in the design and construction of cellular systems for the robust and green conversion of lignocellulose into valuable products.

## Data availability statement

The datasets presented in this study can be found in online repositories. The names of the repository/repositories and accession number(s) can be found in the article/Supplementary material.

## Author contributions

MY: Formal analysis, Investigation, Methodology, Software, Visualization, Writing – original draft. QZ: Formal analysis, Investigation, Methodology, Software, Visualization, Writing - original draft. YL: Formal analysis, Writing – review & editing. WZ: Writing – review & editing, Data curation, Software. ZS: Writing – review & editing, Formal analysis, Visualization. ZR: Writing – review & editing, Investigation, Methodology, Validation, Writing – original draft. CX: Visualization, Writing – review & editing, Methodology, Formal analysis, Conceptualization, Funding acquisition, Investigation, Writing – original draft.

## Footnotes

^1^ https://www.ncbi.nlm.nih.gov/

^2^ https://ftp.ncbi.nih.gov/pub/COG/COG2020/data/

^3^ https://www.kegg.jp/blastkoala/

^4^ https://pfam.xfam.org/
