# Supplementary material for: Insights into lignocellulose degradation: comparative genomics of anaerobic and cellulolytic Ruminiclostridium-type species
Source: Front Microbiol. 2023 Nov 23;14:1288286. doi: 10.3389/fmicb.2023.1288286 (PMC10701907; doi:10.3389/fmicb.2023.1288286)
Supplement: Supplementary file 1 [file Table_1.DOCX]

**Table S1. The general features of the *Clostridia* species genome.**

| Species_name | GenBank Accession Number | Size(Mb) | GC% | Scaffolds | Gene | Proteins | Assembly Level | Reference |
| --- | --- | --- | --- | --- | --- | --- | --- | --- |
| Clostridium acetobutylicum ATCC824 | AE001437.1 | 4.1 | 30.5 | 1 | 3,974 | 3,815 | Complete | (Nolling et al., 2001) |
| Clostridium cellulovorans 743B | CP002160.1 | 5.26 | 31.2 | 1 | 4,492 | 4,313 | Complete | (Sleat et al., 1984) |
| Ruminiclostridium herbifermentans MA18 | CP061336.1 | 4.82 | 33.2 | 1 | 3,838 | 3,701 | Complete | (Rettenmaier et al., 2019) |
| Ruminiclostridium sufflavum DSM19573 | QKMR00000000.1 | 4.4 | 38.7 | 57 | 3,757 | 3,631 | Scaffold | (Nishiyama et al., 2009) |
| Ruminiclostridium hungatei DSM14427 | MZGX00000000.1 | 4.9 | 42.3 | 100 | 4,340 | 4,199 | Contig | (Monserrate et al., 2001) |
| Ruminiclostridium cellobioparum DSM1351 | JHYD00000000.1 | 6.13 | 41.4 | 80 | 5,199 | 5,057 | Scaffold | (Mukherjee et al., 2017) |
| Ruminiclostridium termitidis CT1112 | AORV00000000.1 | 6.42 | 41.2 | 78 | 5,392 | 5,249 | Contig | (Lal et al., 2013) |
| Ruminiclostridium cellulolyticum H10 | CP001348.1 | 4.07 | 37.4 | 1 | 3,534 | 3,383 | Complete | (Desvaux, 2005) |
| Ruminiclostridium josui JCM17888 | JAGE00000000.1 | 4.47 | 36.1 | 2 | 3,947 | 3,785 | Contig | (Sukhumavasi et al., 1988) |
| Ruminiclostridium papyrosolvens C7 | ATAY00000000.1 | 4.4 | 37.1 | 104 | 3,968 | 3,843 | Contig | (Zepeda et al., 2013) |
| Ruminiclostridium sp. BNL1100 | CP003259.1 | 4.61 | 37.3 | 1 | 4,067 | 3,933 | Complete | (Li et al., 2012) |
| Ruminiclostridium papyrosolvens DSM2782 | CP119677.1 | 5.03 | 37.09 | 1 | 4,407 | 4,274 | Complete | (Zou et al., 2018) |

DESVAUX, M. 2005. Clostridium cellulolyticum: model organism of mesophilic cellulolytic clostridia. *FEMS Microbiol Rev,* 29**,** 741-64.

LAL, S., RAMACHANDRAN, U., ZHANG, X., MUNIR, R., SPARLING, R. & LEVIN, D. B. 2013. Draft Genome Sequence of the Cellulolytic, Mesophilic, Anaerobic Bacterium Clostridium termitidis Strain CT1112 (DSM 5398). *Genome Announc,* 1.

LI, L. L., TAGHAVI, S., IZQUIERDO, J. A. & VAN DER LELIE, D. 2012. Complete genome sequence of Clostridium sp. strain BNL1100, a cellulolytic mesophile isolated from corn stover. *J Bacteriol,* 194**,** 6982-3.

MONSERRATE, E., LESCHINE, S. B. & CANALE-PAROLA, E. 2001. Clostridium hungatei sp. nov., a mesophilic, N2-fixing cellulolytic bacterium isolated from soil. *Int J Syst Evol Microbiol,* 51**,** 123-132.

MUKHERJEE, S., SESHADRI, R., VARGHESE, N. J., ELOE-FADROSH, E. A., MEIER-KOLTHOFF, J. P., GOKER, M., COATES, R. C., HADJITHOMAS, M., PAVLOPOULOS, G. A., PAEZ-ESPINO, D., YOSHIKUNI, Y., VISEL, A., WHITMAN, W. B., GARRITY, G. M., EISEN, J. A., HUGENHOLTZ, P., PATI, A., IVANOVA, N. N., WOYKE, T., KLENK, H. P. & KYRPIDES, N. C. 2017. 1,003 reference genomes of bacterial and archaeal isolates expand coverage of the tree of life. *Nat Biotechnol,* 35**,** 676-683.

NISHIYAMA, T., UEKI, A., KAKU, N. & UEKI, K. 2009. Clostridium sufflavum sp. nov., isolated from a methanogenic reactor treating cattle waste. *Int J Syst Evol Microbiol,* 59**,** 981-6.

NOLLING, J., BRETON, G., OMELCHENKO, M. V., MAKAROVA, K. S., ZENG, Q., GIBSON, R., LEE, H. M., DUBOIS, J., QIU, D., HITTI, J., WOLF, Y. I., TATUSOV, R. L., SABATHE, F., DOUCETTE-STAMM, L., SOUCAILLE, P., DALY, M. J., BENNETT, G. N., KOONIN, E. V. & SMITH, D. R. 2001. Genome sequence and comparative analysis of the solvent-producing bacterium Clostridium acetobutylicum. *J Bacteriol,* 183**,** 4823-38.

RETTENMAIER, R., KOWOLLIK, M. L., KLINGL, A., LIEBL, W. & ZVERLOV, V. 2019. Ruminiclostridium herbifermentans sp. nov., a mesophilic and moderately thermophilic cellulolytic and xylanolytic bacterium isolated from a lab-scale biogas fermenter fed with maize silage. *Int J Syst Evol Microbiol,* 71.

SLEAT, R., MAH, R. A. & ROBINSON, R. 1984. Isolation and Characterization of an Anaerobic, Cellulolytic Bacterium, Clostridium cellulovorans sp. nov. *Appl Environ Microbiol,* 48**,** 88-93.

SUKHUMAVASI, J., OHMIYA, K., SHIMIZU, S. & UENO, K. 1988. Clostridium josui sp. nov., a cellulolytic, moderate thermophilic species from Thai compost. *International Journal of Systematic and Evolutionary Microbiology,* 38**,** 179-182.

ZEPEDA, V., DASSA, B., BOROVOK, I., LAMED, R., BAYER, E. A. & CATE, J. H. 2013. Draft Genome Sequence of the Cellulolytic Bacterium Clostridium papyrosolvens C7 (ATCC 700395). *Genome Announc,* 1.

ZOU, X., REN, Z., WANG, N., CHENG, Y., JIANG, Y., WANG, Y. & XU, C. 2018. Function analysis of 5'-UTR of the cellulosomal xyl-doc cluster in Clostridium papyrosolvens. *Biotechnol Biofuels,* 11**,** 43.
